# Daytime Land Surface Temperature Extraction from MODIS Thermal Infrared Data under Cirrus Clouds

**DOI:** 10.3390/s150509942

**Published:** 2015-04-28

**Authors:** Xiwei Fan, Bo-Hui Tang, Hua Wu, Guangjian Yan, Zhao-Liang Li

**Affiliations:** 1 State Key Laboratory of Resources and Environment Information System, Institute of Geographic Sciences and Natural Resources Research, Chinese Academy of Sciences, Beijing 100101, China; E-Mails: fanxw.11b@igsnrr.ac.cn (X.F.); tangbh@igsnrr.ac.cn (B.-H.T.); wuhua@igsnrr.ac.cn (H.W.); 2 University of Chinese Academy of Sciences, Beijing 100049, China; 3 Jiangsu Center for Collaborative Innovation in Geographical Information Resource Development and Application, Nanjing 210023, China; 4 State Key Laboratory of Remote Sensing Science, School of Geography, Beijing Key Laboratory for Remote Sensing of Environment and Digital Cities, Beijing Normal University, Beijing 100875, China; 5 Key Laboratory of Agri-informatics, Ministry of Agriculture/Institute of Agricultural Resources and Regional Planning, Chinese Academy of Agricultural Sciences, Beijing 100081, China; 6 ICube, UdS, CNRS, 300 Bld Sebastien Brant, CS10413, Illkirch 67412, France

**Keywords:** cirrus clouds, error correction, generalized split-window algorithm, land surface temperature retrieval, MODIS

## Abstract

Simulated data showed that cirrus clouds could lead to a maximum land surface temperature (LST) retrieval error of 11.0 K when using the generalized split-window (GSW) algorithm with a cirrus optical depth (*COD*) at 0.55 μm of 0.4 and in nadir view. A correction term in the *COD* linear function was added to the GSW algorithm to extend the GSW algorithm to cirrus cloudy conditions. The *COD* was acquired by a look up table of the isolated cirrus bidirectional reflectance at 0.55 μm. Additionally, the slope *k* of the linear function was expressed as a multiple linear model of the top of the atmospheric brightness temperatures of MODIS channels 31–34 and as the difference between split-window channel emissivities. The simulated data showed that the LST error could be reduced from 11.0 to 2.2 K. The sensitivity analysis indicated that the total errors from all the uncertainties of input parameters, extension algorithm accuracy, and GSW algorithm accuracy were less than 2.5 K in nadir view. Finally, the Great Lakes surface water temperatures measured by buoys showed that the retrieval accuracy of the GSW algorithm was improved by at least 1.5 K using the proposed extension algorithm for cirrus skies.

## 1. Introduction

Land surface temperature (LST) is an important parameter because of its control on the upward terrestrial radiation and the energy exchange between the Earth’s surface and the atmosphere [1,2]. Satellite remote sensing offers the only possibility to measure LST over extended regions with high temporal and spatial resolutions [3]. However, because of the complex influences of cirrus clouds on atmospheric radiative transfer and relatively low atmospheric transmittances, the current LST retrieval algorithms using satellite thermal-infrared (TIR) data typically do not consider the influences of cirrus clouds and therefore are only applied for clear-sky conditions. 

Cirrus clouds, which are relatively optically thin, have a global coverage of approximately 20%, with over 60%–70% in the tropics [4], and a thin cirrus layer may be present as much as 80% of the time in tropical regions [5]. It has been noted that globally distributed high and thin cirrus clouds introduce serious retrieval difficulties of atmospheric temperature and humidity profiles and surface geophysical parameters from space-based platforms, owing to the semitransparency of these clouds at visible and infrared wavelengths [6,7,8]. Regarding the effects of cirrus clouds on estimates of sea surface temperature (SST), Xu and Sun [8] indicated an error of 1.5–2.0 K on SST retrieval occurs if clear-sky SST retrieval equations were used in the presence of cirrus. Additionally, Fan *et al.* [9] indicated using simulated data that the maximum LST error raised to approximately 12 K in the vertical view when the generalized split-window (GSW) algorithm was used in LST retrieval under cirrus cloudy conditions. 

To retrieve LST under cirrus-skies, Fan *et al.* [10] proposed a three-channel LST retrieval algorithm with considering the variations in cirrus optical depth (*COD*), cirrus effective radius (*R*), and cirrus top height (*CTH*) [11,12]. However, because the algorithm relies on the mid-infrared channel, the observations in the mid-infrared channel at satellite altitudes during the daytime consist of a combination of reflected radiance due to sun irradiance and emitted radiance from both the surface and the atmosphere and hence cannot be used to retrieve LST during the daytime. Thus, the algorithms aim to retrieve daytime SST or LST under cirrus skies are necessary. Because the GSW algorithm is one of the most efficient and commonly used methods to estimate LST from satellite TIR data with an accuracy of better than 1 K at nadir view [13], the objective of this work is to develop an algorithm to extend the clear-sky-based GSW algorithm to cirrus cloudy conditions with considering the variations of *COD*, *R*, and *CTH*. 

The outline of this paper is as follows: Section 2 presents the data used in this study. Section 3 shows the influences of cirrus on LST retrieval and the extension algorithm to reduce those influences. Results and some analyses are provided in Section 4. Section 5 presents the results for validating the proposed algorithm with field measurements and MODIS satellite data. Conclusions are presented in the final section.

## 2. Data 

### 2.1. Simulated Datasets 

The atmospheric radiative transfer code MODerate resolution atmospheric TRANsmission (MODTRAN) is used to simulate Top of the Atmosphere (TOA) radiances for methodology development. First, 60 clear-sky atmospheric profiles, 54 main land cover types [14] and cirrus optical properties of a general habit mixture (GHM) selected from radiosonde observation databases-Thermodynamic Initial Guess Retrieval (TIGR) database [15], Advanced Spaceborne Thermal Emission Reflection Radiometer (ASTER) spectral library [16], and Ice Cloud Bulk Scattering Models [17,18], respectively, are used in the simulations. The specific procedures for the selection of atmospheric profiles and land cover types from TIGR and ASTER, respectively, and a brief introduction of TIGR, ASTER and GHM are provided by [10]. 

Two simulated datasets, *Data-cirrus* and *Data-clearsky*, are produced to extend the GSW algorithm in cirrus skies, where the differences in the two dataset-estimated LSTs are considered as the influences of cirrus on LST retrieval. *Data-cirrus* contains the TOA radiances of MODIS channels 31–34 (centered at 11.0, 12.0, 13.4, and 13.7 μm) with considering the influences of cirrus clouds, and *Data-clearsky* contains the TOA radiances of split-window channels without considering such influences. As the detection limitation of the MODIS cloud mask products is approximately 0.4 for *COD* for the visible bands [19], namely the conventional clear-sky-based LST retrieval algorithms are commonly misused to retrieve LST under cirrus skies when the *COD* is less than 0.4, the slant path *COD* at 0.55 μm varies from 0.04 to 0.4 in steps of 0.04 in the production of *Data-cirrus*. Additionally, cirrus optical properties with an *R* greater than 15 μm are used in this study due to the lower occurrence probability of small particles in cirrus clouds [20]. Furthermore, the *CTH* are varied from 8 km to 16 km in steps 4 km [11]. To increase the representativeness of the simulations, reasonable variations of day-time LST are varied in a wide range from near surface atmospheric temperature (*T_0_*) minus 5 K to *T_0_* plus 15 K in step of 5 K. By adding cirrus optical properties of different *R* into MODTRAN with *COD* (at 0.55 μm) being 0.04 to 0.4 and *CTH* being 8 km to 16 km, the cirrus influenced TOA radiances in four MODIS TIR channels are produced based on the clear-sky atmospheric profiles and land surface emissivities (LSEs) previously selected from TIGR and ASTER, respectively. Other detailed procedures for the production of *Data-cirrus* are referred to in [10]. The TOA brightness temperatures for the MODIS split-window channels in clear-sky conditions (*Data-clearsky*) are produced similarly as those for *Data-cirrus* for methodology development.

### 2.2. Satellite Data 

Four MODIS products are used in the study: (1) Calibrated Radiances 5-Min L1B Swath 1-km dataset (MOD021KM/MYD021KM) in collection 6; (2) Geolocation Fields 5-Min L1A Swath 1-km dataset (MOD03/MYD03) in collection 6; (3) Clouds 5-Min L2 Swath 1-km dataset (MOD06_L2/MYD06_L2) in collections 5.1 and 6, respectively; and (4) Land Surface Temperature and Emissivity 5-Min L2 Swath 1-km dataset (MOD11_L2/MYD11_L2) in collection 5. Those products are saved in Hierarchical Data Format (HDF) and are available from NASA’s Goddard Space Flight Center (GSFC) Level 1 and Atmosphere Archive and Distribution System (LAADS).

The observed radiances in channels 31–34 of the MOD021KM/MYD021KM dataset at a 1-km spatial resolution are used to acquire the corresponding TOA brightness temperatures for extending the GSW algorithm in cirrus cloudy conditions.

The MOD03/MYD03 dataset contains latitude and longitude, solar zenith and azimuth angles, and satellite viewing zenith and azimuth angles for every pixel at a 1-km spatial resolution. The latitude and longitude data are used to perform geometric corrections for three other 1-km resolution MODIS products used in this study. The solar and satellite viewing geometries are applied to convert cirrus reflectance into *COD*, and the satellite viewing zenith angles (VZAs) are used to determine the coefficients of the proposed extension algorithm. 

The cloud mask products in MOD06_L2/MYD06_L2 are used to select the confident clear-sky pixels to eliminate the influences of other types of clouds on MOD11_L2/MYD11_L2 for method validations. Additionally, the isolated cirrus bidirectional reflectance (ICBR) and cirrus reflectance flag are used to determine the *COD* of cirrus in this study. 

The LSTs are extracted from MOD11_L2/MYD11_L2 to compare with the results of the extended GSW algorithm in cirrus skies, and the corresponding LSEs in split-window channels are used to determine the correction term in the extension algorithm. 

### 2.3. Study Area and Field Measurements

**Figure 1:**
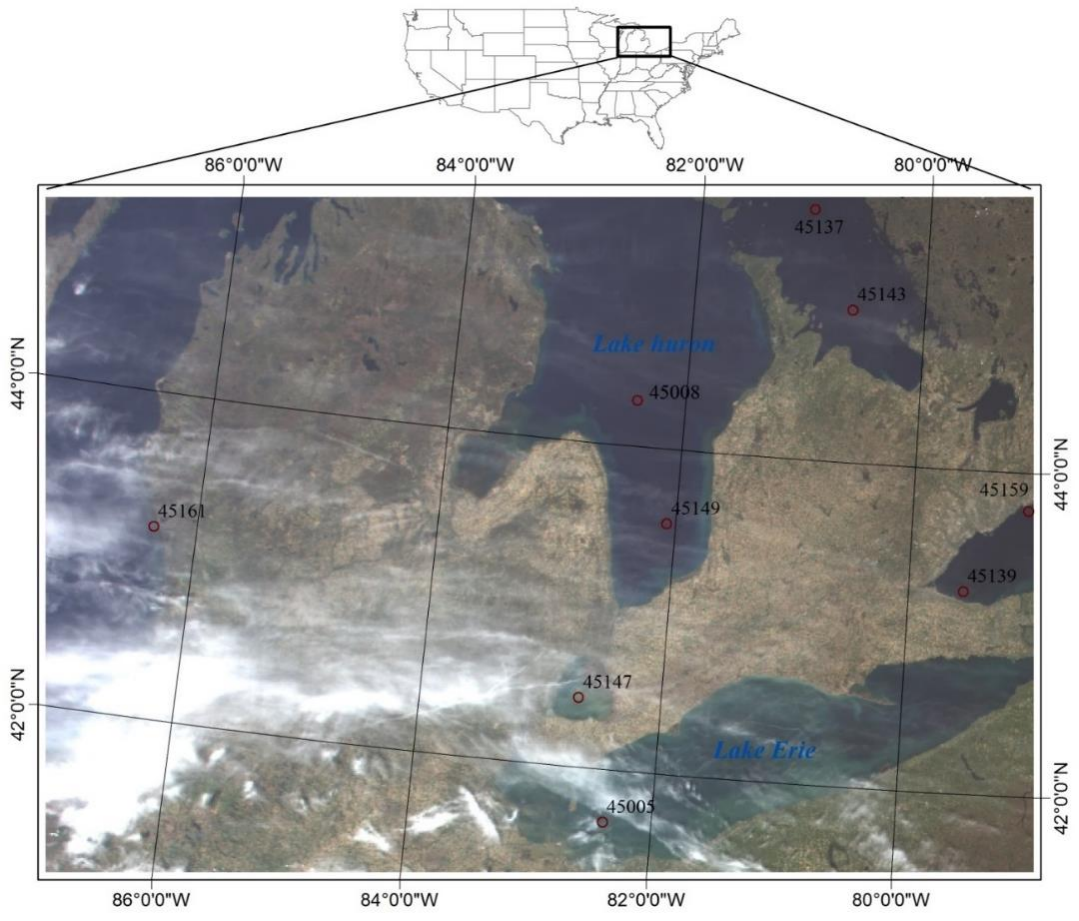
MODIS/Terra true color map of the study area around the Great Lakes on 6 May 2013 (MOD021KM.A2013126.1605.006.2013127012129) indicating the locations of the nine buoys selected in this study.

It is a difficult task to acquire the field measured true values of LST at satellite pixel scales due to the thermal inhomogeneity of the land surface compared with lake or sea surfaces. The water temperatures of the Great Lakes measured with buoys 45005 (41.677°N, 82.398°W), 45008 (44.283°N, 82.416°W), 45161 (43.178°N, 86.361°W) at 0.6 m under water and recorded by the Great Lakes CoastWatch Node (http://coastwatch.glerl.noaa.gov/) are used to represent the daytime MODIS pixel-scale lake surface water temperatures in this study. Additionally, the water temperatures measured with buoys 45137 (45.545°N, 81.015°W), 45139 (43.252°N, 79.535°W), 45143 (44.945°N, 80.627°W), 45147 (42.430°N, 82.683°W), 45149 (43.542°N, 82.075°W), and 45159 (43.767°N, 78.983°W) and recorded by Environment Canada are also used. Figure 1 is the MODIS/Terra true color map of the study area around Great Lakes on 6 May 2013 (MOD021KM.A2013126.1605.006.2013127012129); the locations of the nine buoys are also shown on this map. It can be seen that there are some thick clouds identified by the MODIS cloud mask product at the bottom left of this map; additionally, the thin clouds in the upper part of this map are used to validate the performance of the proposed extension algorithm. 

The lake water temperatures measured by the nine buoys from May to November in 2013 with an accuracy of 0.1 K are collected in this study for algorithm validations. Note that the buoys measure the lake water temperatures with sample intervals of hourly averaged at most, and it meets the recommendations by Minnett [21] that the validation measurements should be made within ±2 h of the satellite overpass. Furthermore, the buoys used in this study are located at least 1 km away from lakeshore to avoid the mixed land/lake surface pixels. 

## 3. Method

### 3.1. LST Retrieval under Cirrus Clouds 

The GSW algorithm extended from the physically based local split-window algorithm proposed by [22] is expressed as [23]:

(1) $$LST=a_{0}\text{+}\left(a_{1}+a_{2}\frac{1-\varepsilon }{\varepsilon }+a_{3}\frac{\Delta \varepsilon }{\varepsilon^{2}}\right)\frac{T_{31}+T_{32}}{2}+\left(a_{4}+a_{5}\frac{1-\varepsilon }{\varepsilon }+a_{6}\frac{\Delta \varepsilon }{\varepsilon^{2}}\right)\frac{T_{31}-T_{32}}{2}$$

where *T*_31_ and *T*_32_ are the TOA brightness temperatures of MODIS split-window channels, respectively; *ε* and Δ*ε* are the mean and difference of the split-window channel emissivities, respectively; the VZA dependent *a*_i_ (*i* = 0–6) are the numerical coefficients of the GSW algorithm. 

**Figure 2:**
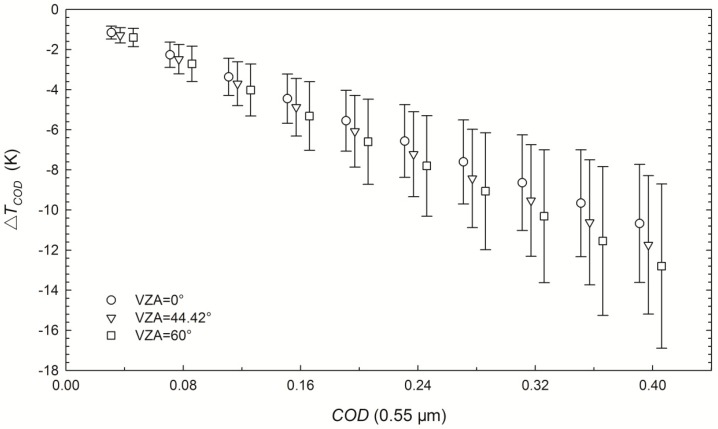
Influence of cirrus clouds on LST retrieval (Δ*T_COD_*) using the GSW algorithm with simulated MODIS data for three VZAs. The hollow symbols indicate the biases and the half-length of vertical bars represent the STDs of Δ*T_COD_* calculated with Equation (2).

To improve the LST retrieval accuracy, the GSW algorithm is divided into six groups according to the water vapor content (WVC) of the atmospheric profiles selected in the previous section with an overlap of 0.5 g/cm^2^: 0–1.5, 1.0–2.5, 2.0–3.5, 3.0–4.5, 4.0–5.5, 5.0–6.5 g/cm^2^ [24]. Then, six groups of *a*_i_ (*i* = 0–6) coefficients in Equation (1) are determined for each VZA using the Levenberg-Marquardt statistical regression method from *Data-clearsky*. The LSTs with (*LST_COD_*) and without (*LST_clear-sky_*) including the influences of cirrus are retrieved from *Data-cirrus* and *Data-clearsky*, respectively, in the GSW algorithm using the coefficients. Note that the errors in *LST_COD_* are the sums of the GSW algorithm errors and the errors caused by cirrus, and the errors in *LST_clear-sky_* are only from GSW algorithm errors, which are irregular for further process. Thus, similar as estimating the influences of dust aerosol on LST retrieval in the GSW algorithm [25], the following equation is used to estimate the influences of cirrus clouds on the retrieval of LST (Δ*T_COD_*):

(2) $$\Delta T_{COD}=LST_{COD}-LST_{clear-sky},\;\;\left(0.04,\ldots ,0.4\right)$$

where Δ*T_COD_* is calculated from the corresponding pairs of *LST_COD_* and *LST_clear-sky_* for each *COD* from 0.04 to 0.4. Figure 2 shows the biases and standard deviations (STDs) of Δ*T*_COD_ versus *COD* at 0.55 μm for three example VZAs, indicating that cirrus clouds lead to an underestimation of the LST retrieved using the GSW algorithm with a bias of −1.2 K with *COD* = 0.04 and VZA = 0°. Both the STD of Δ*T_COD_* and the absolute value of the bias increase as the *COD* increases. The STD and bias reach up to 4.1 K and −12.8 K, respectively, when *COD* = 0.4 and VZA = 60°. 

The biases of Δ*T_COD_* in Figure 2 indicate that the *COD* is one of the primary factors that determine LST retrieval errors because of its dominant influence on cirrus transmittances in split-window channels. Thus, inspired by the extension of the GSW algorithm under dust aerosol skies [25], the LST retrieval accuracy of the GSW algorithm under cirrus skies can be improved using the following correction term:

(3) $$\Delta T_{COD}=k*COD,\;\;\left(COD\leq 0.4\right)$$

where *k* is the slope varying with LSE, *CTH*, *R* and VZA. The offset of Equation (3) is 0 because the LST retrieval errors are 0 without the presence of cirrus. The mean, STD, and maxima of the root mean square errors (RMSEs) between the actual Δ*T_COD_* calculated with Equation (2) and the Δ*T_COD_* regressed based on Equation (3) for the vertical view condition are 0.13, 0.06, and 0.7 K, respectively, which indicates that the proposed linear function can appropriately correct for the LST retrieval error of the GSW algorithm caused by cirrus. Thus, *k* and *COD* are necessary to Equation (3) for extending the GSW algorithm under cirrus skies. 

### 3.2. Determination of Slope k

**Figure 3:**
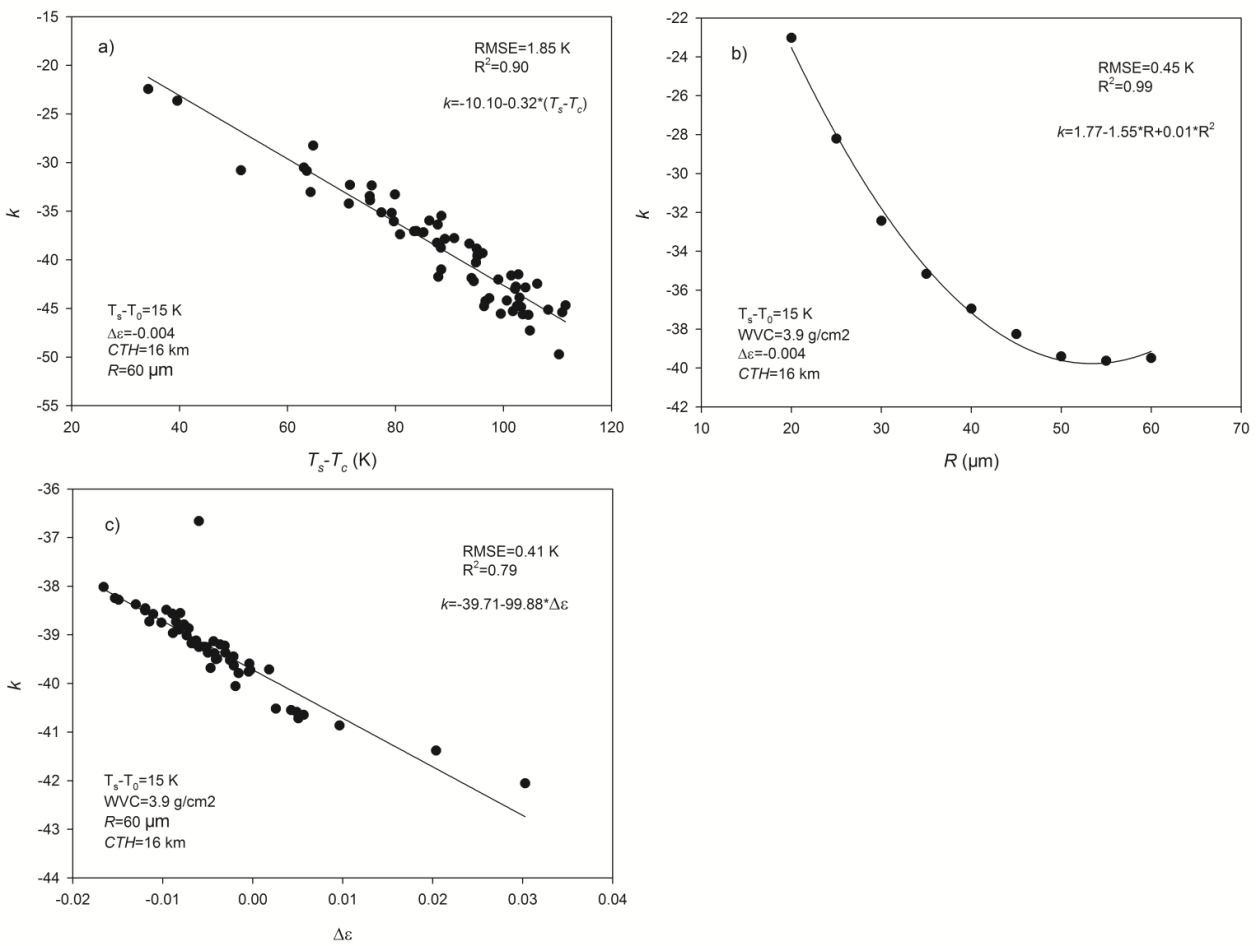
The relationships between slope *k* in Equation (3) and (**a**) the difference between LST and cirrus temperature (*T_s_* − *T_c_*); (**b**) the cirrus effective radius (*R*); (**c**) the difference of the split-window channel emissivities (Δ*ε*).

The determination of slope *k* in Equation (3) is necessary to improve the retrieval accuracy of the GSW algorithm under cirrus skies*.* Because the difference between LST and the cirrus temperature (*T_c_*) is a dominant factor for the reduction in the split-window channel TOA brightness temperatures for a given *COD*, the temperature difference between LST and the cirrus temperature (*T_s_* − *T_c_*) is used to obtain *k*. Additionally, because the cirrus effective radius controls the differences of cirrus transmittances in split-window channels [26], *R* is also employed to calculate *k*. Furthermore, the emissivity difference in split-window channels is also considered to determine *k*. Figure 3 presents the relationships between *k* and *T_s_* − *T_c_*, *R*, and Δ*ε* in the simulated dataset as an example. The slope *k* can be expressed as a linear function of *T_s_* − *T_c_* and Δ*ε* and a quadratic function of *R*. Considering the effects of those factors, a multiple regression model is used to determine the value of *k*:

(4) $$k=31.79-0.26*\left(T_{s}\text{-}T_{c}\right)-2.02*R+0.02*R^{2}-85.22\Delta \varepsilon$$

The coefficients in Equation (4) are determined from the previously simulated dataset in nadir view with the Levenberg-Marquardt statistical regression method. The RMSE between the estimated *k* and the actual value is 3.2 K, and the coefficient of determination (R^2^) of the fitting equation is 0.92, indicating that Equation (4)-calculated *k* can be used to correct for the influences of cirrus on LST retrieval with Δ*T_COD_* – k*COD and after correction the RMSE of Δ*T_COD_* becomes approximately 1.3 K (3.2 K × 0.4) in nadir view when the *COD* at 0.55 μm is 0.4. Additionally, because the *a_i_* (*i* = 0–6) coefficients of the GSW algorithm in Equation (1) depend on the VZA, the biases in Figure 2 are affected slightly by VZA. Therefore, the VZA must also be considered in the determination of slope *k* when correcting for Δ*T_COD_*.

Considering MODIS channels 33 and 34 are commonly related with atmospheric temperature profiles [27], the TOA brightness temperature differences between channels 31 and 34 (*T*_31_ − *T*_34_) and between 31 and 33 (*T*_31_ − *T*_33_) are employed to substitute *T_s_* − *T_c_*. Because the differences in the TOA brightness temperatures in split-window channels are related to the effective radius of cirrus [26], *T*_31_ *−* *T*_32_ is also employed to estimate *k*. Thus, a new multiple linear regression model is used to determine the value of *k* to replace Equation (4):

(5) $$k=k_{0}+k_{1}\left(T_{31}\text{-}T_{34}\right)+k_{2}\left(T_{31}\text{-}T_{33}\right)+k_{3}\left(T_{31}\text{-}T_{32}\right)+k_{4}\Delta \varepsilon$$

where *k_i_* (*i* = 0–4) are the statistical regression coefficients dependent on VZA, which are shown in Table 1. The RMSEs between the estimated and actual *k* for different VZAs are also shown in Table 1. The minimum and maximum RMSEs of Equation (5)-estimated *k* are 5.67 and 8.33 K for VZA = 0° and 60°, respectively, leading to LST errors of 2.26 and 3.33 K when *COD* is 0.4. These results indicate that after *T_s_* − *T_c_* and *R* are substituted with *T*_31_ − *T*_34_, *T*_31_ − *T*_33_, and *T*_31_ − *T*_32_, the LST errors after correction increase by approximately 1 K when VZA = 0°. The value of *k_i_* (*i* = 0–4) can be interpolated by secant VZA for the VZAs not included in Table 1. 

**Table 1:** Values of coefficients *k_0_*, *k_1_*, *k_2_*, *k_3_* and *k_4_* in Equation (5) for six VZAs.

Secant(VZA)	*k_0_* (K)	*k_1_*	*k_2_*	*k_3_*	*k_4_* (K)	RMSE (K)
1.0	−17.57	0.67	−1.39	−1.09	−37.85	5.67
1.2	−20.38	0.97	−1.73	−1.48	−27.90	6.41
1.4	−21.37	0.92	−1.58	−2.21	−13.11	7.01
1.6	−22.28	0.92	−1.51	−2.74	−3.18	7.47
1.8	−22.86	0.89	−1.44	−3.18	4.47	7.87
2.0	−22.84	0.72	−1.16	−3.8	12.92	8.33

### 3.3. Determination of COD

The MODIS 1.375-μm channel is a strong water vapor absorption band, and the reflectance essentially is caused by the reflection and scattering of cirrus clouds because of the relatively small attenuation by the water vapor above and within cirrus and small sensitivity to the lower level water clouds and to the surface. Using the 1.375 μm “cirrus detection” band, the ICBR at visible wavelength, what is free of surface reflection and atmospheric effects and is independent of cloud mask algorithm, is produced as part of MODIS cloud products-MOD06_L2/MYD06_L2 [28].

**Figure 4:**
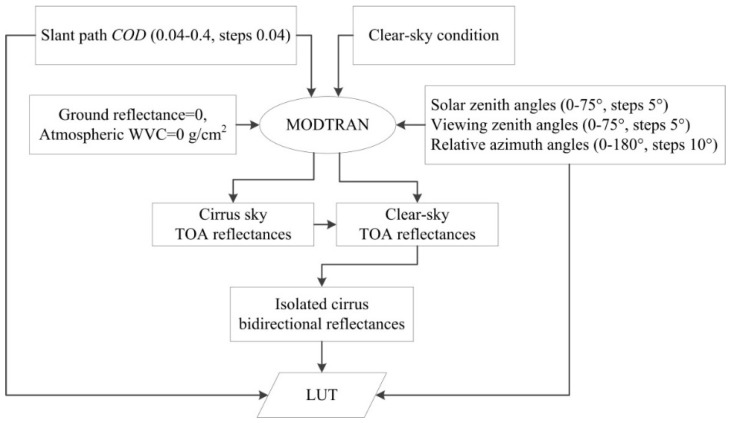
Procedure for the generation of the ICBR LUT.

A look up table (LUT) of the ICBR at different *COD*, solar and satellite viewing geometries are constructed with MODTRAN to acquire *COD*. A total of 4864 solar and satellite viewing geometries in this LUT are considered with solar and satellite VZAs increasing from 0° to 75° at intervals of 5° and relative azimuth angles varying from 0° to 180° at intervals of 10°. Additionally, because the cloud reflectance in the visible channel is sensitive to *COD* but insensitive to *R* [29], the cirrus optical properties when *R* is 20 μm in the GHM is used in the simulation with a slant path *COD* at 0.55 μm varying from 0.04 to 0.4 in steps of 0.04. The atmospheric WVC and surface reflectance are both 0 in the simulation to eliminate the influences of the atmosphere and the land surface. The differences in the TOA reflectance at 0.55 μm between cirrus-sky and clear-sky conditions then are taken as the ICBR. The *COD* can be determined with the inversion of the LUT with the ICBR at given solar and satellite viewing geometries, and the cirrus cloudy pixels can be discriminated from actual clear-sky pixels simultaneously with a *COD* greater than 0.02 [30] and a cirrus reflectance flag of “cirrus”. The procedure for the construction of this LUT is shown in Figure 4.

## 4. Results and Analysis

### 4.1. Results 

**Figure 5:**
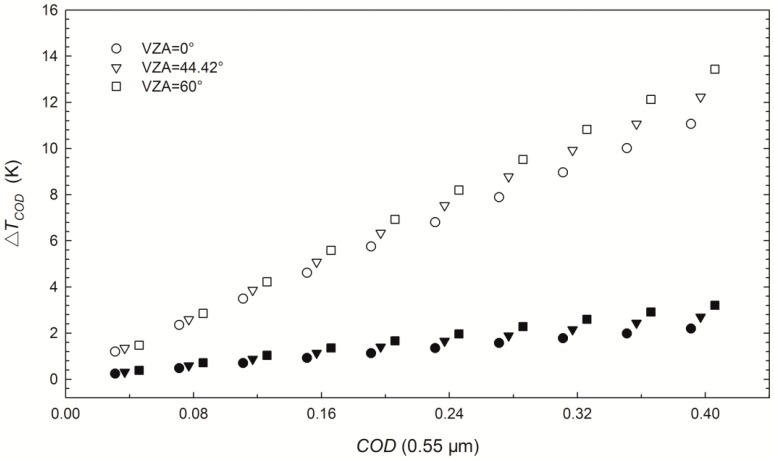
RMSEs between the *LST_clear-sky_* and *LST_COD_* (hollow symbols) and the RMSEs between the *LST_clear-sky_* and *LST_AC_* (solid symbols) for three VZAs. *LST_COD_* and *LST_clear-sky_* are those retrieved from *Data-cirrus* and *Data-clearsky* using the GSW algorithm, respectively. *LST_AC_* are the LSTs of *LST_COD_* minus the correction term k*COD.

Using the *COD* that can be interpolated from the ICBR LUT in actual applications, and with the slope *k* determined from TOA brightness temperatures in MODIS channels 31–34 and split-window channel emissivities, the LST retrieve accuracy of the GSW algorithm under cirrus skies is improved using the correction term *LST_COD_* − k*COD = *LST_AC_*. Figure 5 shows the RMSEs between the *LST_COD_* and *LST_clear-sky_* (hollow symbols) and the RMSEs between the *LST_AC_* and *LST_clear-sky_* (solid symbols). The RMSE is 1.2 K with a *COD* of 0.04 and a VZA of 0°, increasing to 13.4 K with a *COD* of 0.4 and a VZA of 60° without correction. Figure 5 also indicates that the RMSE increases slightly with increasing VZA and *COD* after correction; the maximum RMSE between *LST_AC_* and *LST_clear-sky_* is 3.2 K when the *COD* at 0.55 μm is 0.4 and a VZA of 60°. 

**Figure 6:**
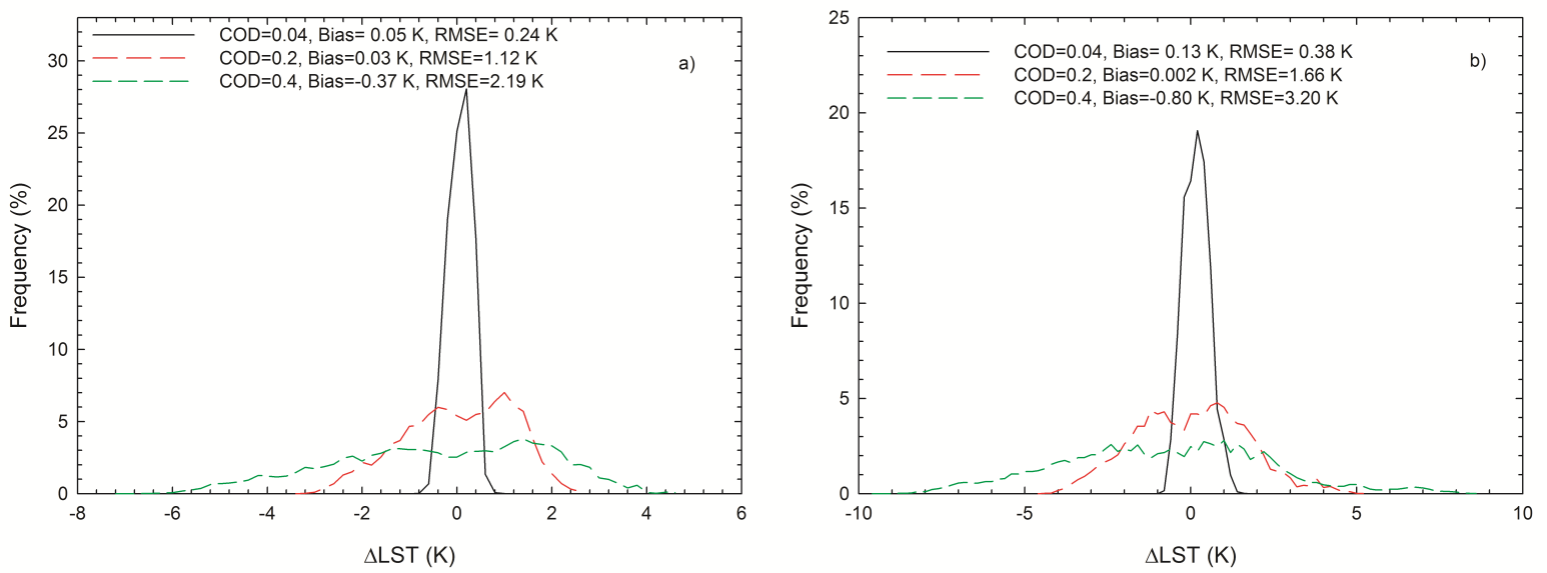
Histograms of LST errors after correction (ΔLST = Δ*T_COD_* − k*COD) with a *COD* (at 0.55 μm) = 0.04, 0.2 and 0.4 for (**a**) VZA = 0° and (**b**) VZA = 60°.

Figure 6 shows histograms of ΔLST from ΔLST = Δ*T_COD_* − k*COD when the *COD* at 0.55 μm are 0.04, 0.2, and 0.4, respectively. The ΔLST is mainly between ±1 K when the *COD* is 0.04. The ΔLST varies between −6 and 4 K for VZA = 0° with a *COD* of 0.4 and between −7 and 5 K for VZA = 60°. The bias is −0.8 K after correction compared to −12.8 K before correction when *COD* is 0.4 and VZA is 60°. It is clear from this figure that the proposed extension algorithm can improve the retrieval accuracy of the GSW algorithm under cirrus skies significantly. 

### 4.2. Analysis

The uncertainty of the proposed GSW extension algorithm for cirrus cloudy conditions is evaluated with a sensitivity analysis. Additionally, the total errors of the extended GSW algorithm-retrieved LST (δ(*LST_AC_*)) is a combination of the extension algorithm accuracy (δ(*LST*_A_)), the errors associated with the uncertainties of the input parameters (δ(*LST_P_*)), and the accuracy of the GSW algorithm (δ(*LST_GSW_*)):

(6) $$\delta \left(LST_{AC}\right)=\sqrt{\delta {\left(LST_{A}\right)}^{2}+\delta {\left(LST_{P}\right)}^{2}+\delta {\left(LST_{GSW}\right)}^{2}}$$

with:

(7) $$\delta \left(LST_{P}\right)=\sqrt{\delta {\left(T_{34}\right)}^{2}+\delta {\left(T_{33}\right)}^{2}+\delta {\left(T_{32}\right)}^{2}+\delta {\left(T_{31}\right)}^{2}+\delta {\left(\Delta \varepsilon \right)}^{2}+\delta {\left(COD\right)}^{2}}$$

where:

(8) $$\delta \left(T_{i}\right)=\left|\frac{\partial \Delta T_{COD}}{\partial T_{i}}\Delta T_{i}\right|,\;\;\left(i=31,32,33,\;or\;34\right)$$

(9) $$\delta \left(\Delta \varepsilon \right)=\left|\frac{\partial \Delta T_{COD}}{\partial \Delta \varepsilon }\Delta \varepsilon \right|$$

(10) δ(COD)=|k*Δ(COD)|

where Δ*T_i_* (*i* = 31, 32, 33, or 34), Δ(Δ*ε*), and Δ(*COD*) are the uncertainties of *T_i_*, Δ*ε*, and *COD*, respectively; the δ(*T_i_*) (*i* = 31, 32, 33, or 34), δ(Δ*ε*), and δ(*COD*) are the LST errors caused by Δ*T_i_*, Δ(Δ*ε*), and Δ(*COD*), respectively.

**Figure 7:**
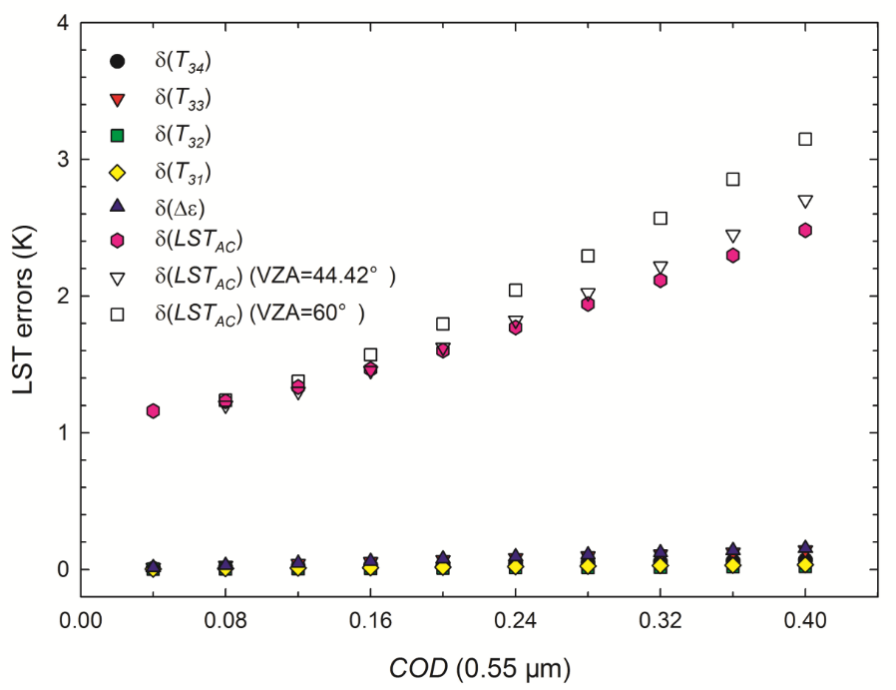
LST errors of δ(*T_i_*) (*i* = 31, 32, 33, and 34) and δ(Δ*ε*) caused by the uncertainties of *T_i_* and Δ*ε*, respectively, and the total LST errors (δ(*LST_AC_*)) in vertical view *versus* *COD* at 0.55 μm (solid symbols). δ(*LST_AC_*) when VZA = 44.42° and 60° is shown in hollow triangles and hollow rectangles, respectively.

The RMSEs between *LST_AC_* and *LST_clear-sky_* as the solid symbols show in Figure 5 are taken as δ(*LST*_A_). The accuracy of the *COD* (Δ(*COD*)) derived from cirrus reflectance is approximately 0.02 [30]. Because the LST errors from the uncertainties of *COD* are multiplied by *k* as in Equation (10), the mean slope of the biases versus *COD* in Figure 2 is used to determine δ(*COD*). Then, the δ(*COD*) are 0.53 and 0.61 K for VZA = 0° and 60°, respectively. Considering MODIS instrument errors of 0.25 K for channels 34 and 33 and 0.05 K for channels 32 and 31, a typical error of 0.01 for Δ*ε*, and 1 K for δ(*LST_GSW_*) [13], the δ(*T_i_*) (*i* = 31, 32, 33, or 34), δ(Δ*ε*), and δ(*LST_AC_*) in vertical view are shown in Figure 7 with solid symbols. Additionally, the δ(*LST_AC_*) when VZA = 44.42° and 60° are also shown in Figure 7 with hollow triangles and hollow rectangles, respectively.

The LST errors δ(*T_31_*), δ(*T_32_*), δ(*T_33_*), δ(*T_34_*), and δ(Δ*ε*) in Figure 7 as a result of the uncertainties in *T_31_*, *T_32_*, *T_33_*, *T_34_*, and Δ*ε* are all less than 0.2 K, indicating that the LST errors from the uncertainties of *COD* are larger compared with the errors from other input parameter uncertainties. Additionally, the total LST errors δ(*LST_AC_*) increase with increases in *COD* because the error of the extension algorithm is the dominant factor in the total LST errors. The maxima of δ(*LST_AC_*) are 2.5 and 3.4 K for VZA = 0° and 60°, respectively. 

Because the constant coefficients in the GSW algorithm cannot be used for worldwide LST retrievals under various atmospheric profiles [22] or land surface conditions, the GSW algorithm is commonly divided into several sub-ranges according to atmospheric WVC, LSE, *T_0_* (or LST). However, the atmospheric WVC estimates may be inaccurate because of the changes in the TOA reflectance of the atmospheric WVC retrieval bands due to the presence of cirrus [31]. In addition to the uncertainties discussed above, the LST errors as a result of the misapplication of the GSW algorithm due to inaccurate input of atmospheric WVC is not considered in this study. 

*COD* is a vital factor in identifying and correcting for LST errors in the GSW algorithm under cirrus skies. However, the accuracy of *COD* may decrease in some extreme dry atmospheric conditions, such as over desert areas, because the MODIS “cirrus detection” channel can be contaminated slightly by lower level water clouds and bright surfaces. Thus, the performance of the extension algorithm in those regions may not be as accurate as in humid atmospheric regions or on dark surfaces, such as over lake or sea surfaces. Fortunately, the probability of cirrus occurrence is low in dry atmospheres [11]

The values of *T_s_* *−* *T_c_* and *R* are substituted with a multiple linear model of TOA brightness temperatures of MODIS channels 31 to 34 to acquire the slope *k* for actual applications. The accuracy of the extension algorithm decreases by approximately 1 K when Equation (5) is used to determined *k* compared with using the Equation (4)-calculated *k*. The difference in cirrus transmittances in split-window channels is one of the dominant factors in the difference in corresponding channel TOA brightness temperatures and is determined by the cirrus effective radius *R*. To acquire more accurate LST under cirrus-skies, the values of *R* or a more accurate substitute for *R* for the extended GSW algorithm can be used in the following on study. 

## 5. Validations

Assuming the buoy-measured lake water temperatures (*LST_buoy_*) are equivalent to the lake surface water temperatures, the MODIS LST uncertainties can be computed as the differences between LST products-MOD11_L2/MYD11_L2 and *LST_buoy_*. The *COD* is interpolated with the LUT constructed in the previous section using the ICBR in MOD06_L2/MYD06_L2 and the solar and satellite viewing geometries in MOD03/MYD03 to improve the retrieval accuracy of the GSW algorithm under cirrus skies using the extension algorithm. Additionally, *k* is determined from TOA brightness temperatures calculated from MOD021KM/MYD021KM of channels 31–34 and with split-window channel emissivities extracted from MOD11_L2/MYD11_L2. The pixels labeled as confident clear sky in MOD06_L2/MYD06_L2, interpolated *COD* greater than 0.02 and the cirrus reflectance flag as “cirrus” are considered as cirrus-sky pixels to eliminate the influences from other cloud types; the differences between MOD11_L2/MYD11_L2 and *LST_buoy_* are determined then as the LST errors caused by cirrus that can be reduced using the correction term of MOD11_L2/MYD11_L2 minus k*COD (*LST_AC_*). 

**Figure 8:**
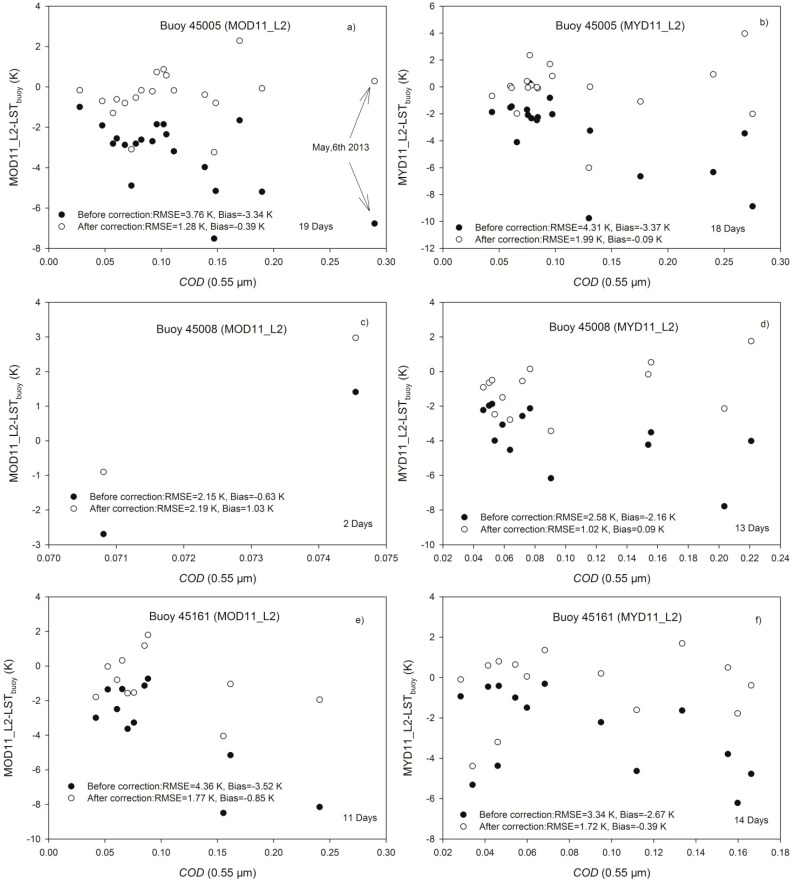
LST differences between LSTs extracted from MOD11_L2/MYD11_L2 and *LST_buoy_* (solid circles) and between the *LST_AC_* and *LST_buoy_* (hollow circles) versus *COD* (at 0.55 μm) for three different buoys from May to November 2013. (**a**,**b**) show the result of MOD11_L2/MYD11_L2 at buoy 45005, respectively; (**c**,**d**) show the result of MOD11_L2/MYD11_L2 at buoy 45008, respectively; (**e**,**f**) show the result of MOD11_L2/MYD11_L2 at buoy 45161, respectively.

Figure 8 shows the LST differences between LSTs extracted from MOD11_L2/MYD11_L2 and *LST_buoy_* (solid circles) and between the *LST_AC_* and *LST_buoy_* (hollow circles) versus *COD* at 0.55 μm for buoys 45005, 45008, and 45161, respectively. The MODIS LSTs retrieved using the clear-sky-based GSW algorithm tend to be underestimated when *COD* increases, and the retrieval accuracy of the GSW algorithm under cirrus skies can be improved using the correction term. The RMSEs of MODIS LSTs at buoy 45005 are 1.28 and 1.99 K after correction for MOD11_L2 and MYD11_L2, respectively, compared with the RMSEs of 3.76 and 4.31 K before correction; similar results are also shown in Figure 8c–f for buoys 45008 and 45161. Except for the MOD11_L2 at buoy 45008 (not considered because it includes only two days), the RMSEs of MODIS LSTs after correction are less than half of the original RMSEs before correction. The proposed algorithm can improve the retrieval accuracy of the GSW algorithm under cirrus skies by at least 1.5 K for buoy 45008 with MYD11_L2 data, and the maximum improvement is 2.6 K for buoy 45161 with MOD11_L2 data. The correction results of the remaining six buoys are shown in Table 2.

Because some of the buoys were malfunctioning from May to November in 2013, a majority of the available validation samplings are less than 5 days (except 45143/MYD11_L2 has 9 days) for the six buoys. Seven of the RMSEs or biases (eleven in all) in Table 2 decrease after correction compared with the RMSEs without correction; however, the remaining four RMSEs tend to increase. The RMSEs are unstable and can be influenced heavily by the correction performance of individual samples due to the small number of available validation samplings. Thus, the validation results in Table 2 do not precisely reflect the performance of the proposed extension algorithm in actual applications.

**Table 2:** Biases and RMSEs of LST errors before and after error correction for buoys 45137, 45139, 45143, 45147, 45149, and 45159. The data are collected from May to November 2013.

Data	Days	MOD11_L2/ MY11_L2		*LST_AC_*	
		Bias (K)	RMSE (K)	Bias (K)	RMSE (K)
45137/MOD11_L2	0	-	-	-	-
45137/MYD11_L2	5	−2.94	3.49	2.05	4.68
45139/MOD11_L2	4	−4.71	5.67	−3.37	4.88
45139/MYD11_L2	3	−3.19	3.26	−0.35	1.10
45143/MOD11_L2	3	−2.10	2.28	1.71	3.40
45143/MYD11_L2	9	−1.54	1.67	1.55	3.44
45147/MOD11_L2	5	−5.77	6.05	0.35	2.34
45147/MYD11_L2	1	−1.95	-	1.36	-
45149/MOD11_L2	1	−4.09	-	0.52	-
45149/MYD11_L2	2	−0.93	1.75	−0.09	1.24
45159/MOD11_L2	1	−2.85	-	4.20	-
45159/MYD11_L2	3	−2.76	3.52	−1.15	1.89

**Figure 9:**
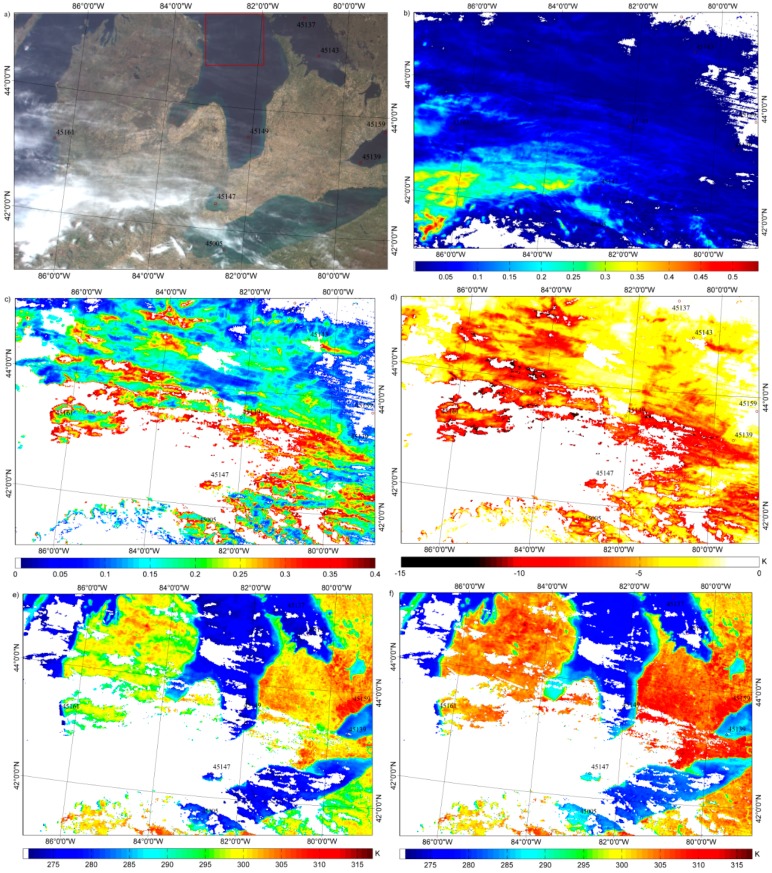
(**a**) Terra/MODIS true color map of the study area, (**b**) ICBR in MOD06_L2, (**c**) *COD* at 0.55 μm where the *COD* is greater than 0.02 and less than 0.4, the cirrus reflectance flags are “cirrus”, and the corresponding LSTs in MOD11_L2 are available, (**d**) LST errors estimated using the proposed extension algorithm (k*COD), (**e**) MOD11_L2, and (f) MOD11_L2 corrected with k*COD.

For example, 6 May 2013 in Figure 9 shows the (a) Terra/MODIS true color map of the study area, (b) ICBR in MOD06_L2, (c) *COD* at 0.55 μm where the *COD* is greater than 0.02 and less than 0.4, the cirrus reflectance flags are “cirrus”, and the corresponding LSTs in MOD11_L2 are available, (d) LST errors estimated using the proposed extension algorithm (k*COD), (e) MOD11_L2, and (f) MOD11_L2 after correction (*LST_AC_*). The maximum value of the LSTs increases from 313 K in MOD11_L2 to 317 K for *LST_AC_* after correction, and the surface water temperatures of Lake Huron shown in the red rectangle (Figure 9a) are more homogeneous after correction (Figure 9f) compared with MOD11_L2 (Figure 9e). Eight buoy-measured lake water temperatures when MODIS/Terra observed on 6 May 2013 are shown in Table 3 to study the correction performance of the proposed extension algorithm shown in Figure 9. The corresponding pixel *COD* at 0.55 μm, the lake surface water temperatures in MOD11_L2 and *LST_AC_* are also shown in Table 3. Because there is no LST value in MOD11_L2 at buoy 45008, its result is no shown in this Table. The *LST_AC_* tend to be more close to *LST_buoy_* compared with MOD11_L2. The RMSE and bias between *LST_AC_* and *LST_buoy_* in Table 3 are 1.2 and 0.4 K, respectively and 5.5 and −4.0 K between MOD11_L2 and *LST_buoy_*. 

**Table 3:** Buoy locations and buoy-measured lake water temperature (*LST_buoy_*) for MODIS/Terra observations on 6 May 2013; cirrus optical depth (*COD*) at 0.55 μm, MODIS/Terra LST products-MOD11_L2, and MOD11_L2 after correction (*LST_AC_*) for the pixels where the buoys are located.

Buoy	Latitude (°)	Longitude (°)	*LST_buoy_* (K)	*COD*	MOD11_L2 (K)	*LST_AC_* (K)
45005	41.677	−82.398	282.45	0.28	275.68	282.74
45137	45.545	−81.015	276.85	0	276.90	276.90
45139	43.252	−79.535	284.25	0.14	282.22	285.80
45143	44.945	−80.627	276.55	0.19	273.14	277.50
45147	42.430	−82.683	286.85	0.39	276.10	287.03
45149	43.542	−82.075	276.55	0.16	275.40	278.96
45159	43.767	−78.983	281.45	0	281.40	281.40
45161	43.178	−86.361	282.25	0.24	274.10	280.34

Additionally, the *COD* at buoys 45137 and 45159 are both 0 where the LSTs in MOD11_L2 are the same as the corresponding LSTs in *LST_AC_*, where the biases of MOD11_L2 at the corresponding buoys are 0.05 and −0.05 K, respectively. This result indicates that the proposed extension algorithm can improve the retrieval accuracy of the GSW algorithm in cirrus skies and can discriminate actual clear-sky conditions from cirrus skies accurately.

**Figure 10:**
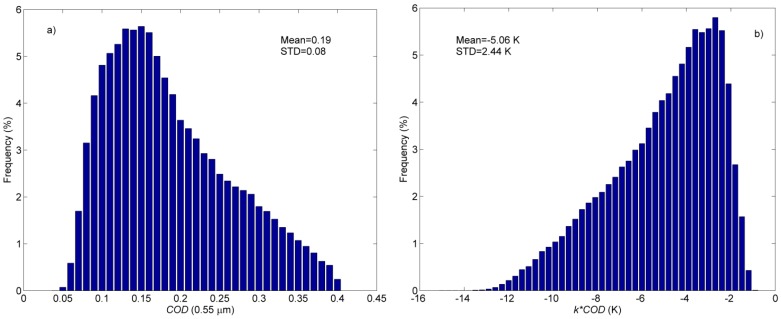
Histograms of (**a**) *COD* at 0.55 μm in Figure 9c and (**b**) LST errors estimated using the proposed extension algorithm (k*COD) in Figure 9d.

Figure 10a,b shows the histogram of the *COD* in Figure 9c and the histogram of the LST errors k*COD in Figure 9d, respectively. Assuming the correction accuracy (the RMSEs are 5.5 and 1.2 K for MOD11_L2 and *LST_AC_*, respectively) of the eight spatially scattered buoys can represent the correction performance of the available pixels in Figure 9d, then it can also be assumed that the clear-sky-based GSW algorithm is commonly misused for the retrieval of LST in cirrus skies with a mean and STD of cirrus *COD* at 0.55 μm of 0.19 and 0.08, respectively; and as a result, the LSTs are underestimated with the mean and STD of −5.06 and 2.44 K, respectively. Figure 7 indicates that the total LST errors after correction are 1.5 and 2.0 K when VZA = 0° and 60°, respectively, for a *COD* of 0.2. Hence, LSTs with a mean error of 1.5–2.0 K can be acquired under cirrus cloudy conditions using the proposed extended GSW algorithm.

## 6. Conclusions

Conventional LST retrieval algorithms from TIR data are developed and applied for clear-sky conditions, and the influences of cirrus clouds are not considered in their development. To study the influences of cirrus clouds on LST retrieval, two different simulated datasets (with and without including the influences of cirrus clouds) were used to retrieve LSTs using the GSW algorithm with the same coefficients obtained from the later dataset. The differences between the two groups of retrieved LSTs indicated that the maximum RMSE was 11.0 K when the *COD* at 0.55 μm was 0.4 and in nadir view.

An extension algorithm is proposed by adding a correction term in function of *COD* in the GSW algorithm to improve the retrieval accuracy in cirrus cloudy conditions. A LUT of the ICBR at 0.55 μm was established with MODTRAN for different *COD*, solar and satellite viewing geometries to acquire *COD*. Then, the *COD* at 0.55 μm was determined with an inversion of the LUT for a given ICBR in the MODIS cloud products, and the cirrus-sky pixel was discriminated from actual clear-sky simultaneously. Additionally, the slope *k* of the linear function was expressed as a multiple linear model of the TOA brightness temperature differences between MODIS channels 31 and 34 (*T*_31_ − *T*_34_), 31 and 33 (*T*_31_ − *T*_33_), 31 and 32 (*T*_31_ − *T*_32_), and the difference between split-window channel emissivities (Δε). The simulated data indicated that the LST error caused by cirrus was reduced from 11.0 to 2.2 K after correction using the proposed extension algorithm. 

The LST errors caused by the uncertainties in the input variables *T*_34_, *T*_33_, *T*_32_, *T*_31_, Δε, and *COD* were analyzed to study the uncertainty of the proposed extension algorithm in cirrus skies. The LST errors from MODIS instrument noise and Δε were less than 0.2 K, and the errors from *COD* were approximately 0.6 K. The total LST errors of the extended GSW algorithm caused by the uncertainties of the input parameters, extension algorithm accuracy, and the GSW algorithm accuracy were also analyzed. The results showed that the total LST errors increased with increasing *COD*, and the maximum total LST error was 2.5 K for *COD* = 0.4 and VZA = 0°. 

The buoy-measured lake water temperatures in the Great Lakes were used to validate the performance of the proposed extension algorithm for LST retrievals. The validations showed that the proposed extension algorithm could improve the retrieval accuracy of the GSW algorithm by at least 1.5 K under cirrus skies, or more than half of the original RMSE values before correction. 

Note that in addition to MODIS, other satellite TIR data-retrieved LSTs under cirrus skies, such as Suomi-NPP/VIIRS or Landsat-8/(OLI and TIRS) that equipped with a cirrus detection band, can also be corrected using this algorithm even with a constant slope *k*. This algorithm can be used not only for improving LST retrieval accuracy but also to improve the SST-retrieve accuracy under cirrus with a higher accuracy.
